# Effects of age and pasture type on the concentration and prevalence of tetracycline and macrolide resistant *Enterococcus* species in beef cow-calf production system

**DOI:** 10.3389/frabi.2022.1052316

**Published:** 2022-11-03

**Authors:** Getahun E. Agga, Hunter O. Galloway, Annesly M. P. Netthisinghe

**Affiliations:** ^1^ Food Animal Environmental Systems Research Unit, Agricultural Research Service, United States Department of Agriculture, Bowling Green, KY, United States; ^2^ Department of Agriculture and Food Science, Western Kentucky University, Bowling Green, KY, United States

**Keywords:** antimicrobial resistance, macrolide resistance, tetracycline resistance, *enterococcus*, cow-calf, beef cattle

## Abstract

Enterococci are a normal flora of the gastrointestinal tracts of humans and animals. Enterococci can also cause life-threatening nosocomial infections. Antimicrobial-resistant *Enterococcus* species have been reported in the feedlot and dairy cattle productions and in meat and milk products, suggesting their foodborne importance. Cow-calf operations represent a significant segment in the beef production system by producing weaned calves. Weaned calves are brought into the feedlot to be finished for meat, and culled cows are also slaughtered for beef, primarily for ground beef products. Infection dynamics in the cow-calf operation can contribute to meat contamination. This study evaluated the effects of age and wheat grazing on the concentration and prevalence of a macrolide antibiotic erythromycin (ERY^r^) and tetracycline (TET^r^) resistant enterococci, associated resistance genes and species distribution in a cow-calf production system. In 2017 and 2018, 32 Angus breed cow-calf pairs were randomly assigned to feed on tall fescue or wheat pasture in two independent field experiments. During the grazing experiments of 2-3 weeks, fecal samples were collected weekly and cultured to enumerate, isolate and identify ERY^r^, TET^r^, and generic enterococci, using media supplemented with erythromycin, tetracycline or non-supplemented media, respectively. The two main species frequently associated with human illnesses, *Enterococcus faecium* and *E. faecalis*, were widely distributed in the cow-calf groups. Generic and TET^r^- enterococci were prevalent (96-100% prevalence) and abundant (3.2-4.9 log_10_ CFU/g) in the cow-calf population; however, ERY^r^ enterococci were enumerable by direct plating only from a single cow despite being detected in at least 40% of the fecal samples after enrichment, showing their low abundance. TET- and ERY-resistance were mainly conferred by *tet*(M) and *erm*(B), respectively. Wheat grazing reduced the concentration of TET^r^ enterococci and modified enterococcal species and resistance gene distributions. Hence, it is necessary to further investigate wheat grazing in cow-calf production as a potential strategy to mitigate antimicrobial resistance.

## Introduction

Enterococci are commensal bacteria colonizing the gastrointestinal tract (GIT) of healthy humans and animals, without causing intestinal problems. (USDA, 2012; Lebreton et al., 2014; Ahmed and Baptiste, 2018) Enterococci can also cause life threatening extraintestinal infections. (Arias and Murray, 2012; Lebreton et al., 2014; Ahmed and Baptiste, 2018) Consequently, antibiotic resistant enterococci are the leading cause of nosocomial infections in the United States. (Arias and Murray, 2012; Lebreton et al., 2014) *Enterococcus faecalis* and *E. faecium* are major species commonly associated with human infections. (Arias and Murray, 2012; Lebreton et al., 2014; Ahmed and Baptiste, 2018) Enterococci are ubiquitous; (Lebreton et al., 2014) people are colonized when exposed to contaminated environments from human and animal wastewater (Agga et al., 2015), contaminated drinking, and recreational water sources. (Lebreton et al., 2014; Cho et al., 2020; Agga et al., 2022a; Kaiser et al., 2022) Furthermore, enterococci have been reported from retail beef (Tyson et al., 2018), retail veal meat (Tate et al., 2021), and ground beef (Vikram et al., 2018; Schmidt et al., 2021) suggesting their foodborne implications. Antimicrobial resistance of enterococci have been extensively studied in feedlot cattle (Vikram et al., 2017; Davedow et al., 2020; Murray et al., 2022), dairy cows (Shipp and Dickson, 2012; Abdelfattah et al., 2021), and to a lesser extent in cull cows. (Pandit et al., 2021) However, such studies are rare in beef cow-calf (Agga et al., 2016) and backgrounding operations (Agga et al., 2019).

Beef cows are culled from the cow-calf operation and sold for beef production similarly to cull dairy cows with the majority of the beef used for ground beef production. While some female calves are selected as replacements for breeding stock, majority of calves are weaned and enter feedlots to be finished for beef production. Determining the level of antimicrobial resistant bacteria (ARB) in weaned calves prior to entering the feedlot is important. Antimicrobial resistant bacteria such as enterococci can contaminate beef carcasses during slaughter process and pose a significant public health risk. Establishing a baseline prevalence of ARB including enterococci in adult beef cows and beef claves is essential. Previous studies reported a decreasing trend in the prevalence of antimicrobial resistant fecal bacteria, mainly *E. coli*, as animals get older. (Hoyle et al., 2004; Gaire et al., 2021) However, studies assessing the impact of age on Gram-positive bacteria such as enterococci are scarce. Therefore, further understanding of the age effect will be useful to differentially target the adult cows and calves for the mitigation of antimicrobial resistance.

Livestock grazing of cover crops allows producers to gain an immediate economic benefit while reducing input costs. Cover crops provide higher quality forage for livestock as compared to typical native grass pastures. (Franzluebbers, 2007; Poffenbarger, 2010) In the southern Great Plains, it is typical to graze winter wheat fields to give stocker cattle high-quality forage. (Winterholler et al., 2008) Grazing of winter wheat cultivars until the joint stage has been reported to increase grain yield as compared to non-grazed winter wheat. (Redmon et al., 1995) However, no research investigated the impact of wheat grazing on the occurrence of ARB in beef cow-calf production systems in the southern plains. The impact of dual-purpose wheat grazing on grain yield and animal growth performance was reported in the previously published study. (Netthisinghe et al., 2020) In this paper, the impact of wheat grazing on the concentration and prevalence of tetracycline (TET^r^)- and erythromycin (ERY^r^)- resistant enterococci was compared with tall fescue grazing in a cow-calf production system.

Specific objectives of this study were to investigate the effect of grazing pasture type (wheat vs. tall fescue) and age (calf vs. cow) in beef cow-calf production system on the concentration and prevalence of TET^r^ and ERY^r^, enterococcal species, and resistance gene distributions among the resistant strains. An additional objective was to determine the baseline level of antimicrobial resistant enterococci in weaned calves prior to entering the feedlot, and in the breeding cows that would transmit resistant bacteria to the beef calves or culled and used for beef production. Enterococci have been used as indicator organisms for Gram-positive bacteria in antimicrobial resistance (AMR) monitoring systems involving food animal production and animal products (Karp et al., 2017).

Tetracycline resistance was selected because of its abundance and widespread occurrence among various bacterial species and environments (Roberts and Schwarz, 2016) which may be attributed to its highest level of sales for use in food-producing animals. (Tyson et al., 2018; Agga et al., 2022a) Erythromycin is a macrolide that has been investigated with respect to antimicrobial resistant enterococci in beef cattle production (Frye and Jackson, 2013) and retail beef. (Tyson et al., 2018) According to World Health Organization (WHO) categorization of antimicrobials, tetracyclines and macrolides are classified as highly important and highest priority critically important antimicrobial classes for human health, respectively. (Scott et al., 2019) TET^r^ in enterococci develops primarily through ribosomal protection or the efflux of the antibiotic. *tet*(M) and *tet*(L) are the most common tetracycline resistance (*tet*) genes encoding for ribosomal protection and efflux proteins in enterococci, respectively. (Frye and Jackson, 2013; Agga et al., 2022c) The most common acquired resistance mechanism for macrolides is target modification by erythromycin resistance methylase (*erm*) genes, primarily *erm*(B) (Frye and Jackson, 2013).

## Materials and methods

### Experimental design and sample collection

A randomized field trial consisting of two experiments was conducted at Western Kentucky University Agriculture Research and Education Complex in Bowling Green, KY during 2017 and 2018. The study protocol was approved by the Western Kentucky University’s Institutional Animal Care and Use Committee (IACUC# 17-09). The study animals consisting of beef cows and calves close to weaning age were owned and managed by Western Kentucky University. Calves received clostridial vaccine against blackleg, viral respiratory vaccine, and pinkeye vaccine. No other antibiotics were given to the cows or the calves. Detailed description of the cow-calf experiments was previously published (Netthisinghe et al., 2020).

Briefly, in two independent experiments 16 Angus breed cow-calf pairs were equally randomized, blocked on the bodyweight of the calves, to graze on tall fescue or wheat pasture in 2017 and 2018. The cow–calf pairs grazed for three weeks from 21 March to 12 April 2017, or for two weeks from 14 March to 28 March 2018. For the 2017 experiment, fecal grabs or fecal swabs were collected rectally on 21 March (week 0), 28 March (week 1), 04 April (week 2) and 11 April 2017 (week 3). For the 2018 experiment, fecal samples were collected on 14 March (week 0), 21 March (week 1) and 28 March (week 2). Samples were kept on ice and transported to the lab and refrigerated until processed.

### Enumeration and detection of generic-, TET^r^- and ERY^r^-enterococci

Samples were processed and cultured as described. (Agga et al., 2015; Agga et al., 2016; Agga et al., 2022c). Briefly, 10 g of fecal grabs were suspended in 90 mL of buffered peptone water (BPW; Becton, Dickson, and Company [BD], Franklin Lakes, NJ, USA) and homogenized in a laboratory blender. Fecal swabs were suspended in 5 mL BPW and homogenized by centrifugation. After an aliquot was taken for enumeration, the remaining BPW suspension was incubated at 25°C for 2 h, then at 42°C for 6 h and held at 4°C for secondary enrichment.

Generic-, ERY^r^- and TET^r^- enterococci were enumerated on Slanetz and Bartley medium (SBM) agar (Thermo Fisher Scientific, Waltham, MA), SBM plates supplemented with 8 mg/L erythromycin (SBM+ERY), and SBM plates supplemented with 16 mg/L tetracycline (SBM+TET), respectively. To determine prevalence, secondary enrichments were made by transferring 0.5 mL of BPW pre-enrichments to 2.5 mL of enterococcosel broth (ECB; BD), ECB supplemented with 16 mg/L tetracycline (ECB+TET) and ECB supplemented with 8 mg/L erythromycin (ECB+ERY). After incubation at 37°C for 18 to 24 h, ECB cultures were streaked onto SBM, SBM+TET and SBM+ERY plates and incubated overnight at 37°C.

Antibiotics used for selective isolation of resistant strains were obtained from Millipore Sigma (St. Louis, MO), and the Clinical Laboratories Standards Institute (CLSI) resistance breakpoint concentrations (CLSI, 2020) were used.

### PCR confirmation, speciation, and detection of resistance genes

For all plate types, up to two presumptive colonies were inoculated into tryptic soy broth (TSB; BD) and incubated overnight at 37°C. After fresh aliquot was taken for DNA extraction, the remaining broth culture was stored at -20°C after adding 15% glycerol to each well. DNA was extracted from 10 ml of overnight culture by BAX lysis method following the manufacturer’s instructions (DuPont Qualicon, Inc., Wilmington, DE). DNA lysates were used for PCR for confirmation of the genus *Enterococcus*, speciation, and detection of associated resistance genes.

Presumptive enterococci isolates were confirmed by genus specific PCR (Deasy et al., 2000), and *Enterococcus* species were identified by multiplex PCR (Jackson et al., 2004), using published primers and protocols (**Supplementary Table 1**). Phenotypically resistant strains were tested by PCR for the identification of resistance genes. TET^r^ *Enterococcus* isolates were tested for tetracycline resistance (*tet*) genes (**Supplementary Table 2**). ERY^r^ *Enterococcus* species were tested for macrolide, lincosamides and streptogramins (MLS_B_) resistance genes (**Supplementary Table 3**). PCR products were analyzed by capillary gel electrophoresis using the QIAxcel Fast Analysis system (Qiagen, Valencia, CA). All primers used in this study were obtained from Integrated DNA Technologies, Inc. (IDT; Coralville, IA). Representative gel images from capillary electrophoresis for enterococci genus confirmation and species identification, detection of tetracycline resistance genes, and macrolide resistance genes are depicted in **Supplementary Figures 1**–**3**, respectively.

### Data analysis

Enumeration data were compared by animal age (calves vs. cows) and by treatment group (wheat vs tall fescue) using negative binomial regression, and marginal outputs were obtained as log_10_ colony forming units. The prevalence of bacteria and the resistance genes were compared by animal age and pasture type using logistic regression. For both regression analyses, cows and tall fescue served as reference groups. Pairwise contrasts were obtained after adjusting for multiple comparisons by Bonferroni method. Data was analyzed in STATA 16 (StataCorp, College Station, Texas); *P*<0.05 was considered statistically significant.

## Results

### Descriptive analysis

Of the 224 fecal samples, 128 were collected in 2017 and 96 were collected in 2018, equally distributed by animal age (calves and cows) and pasture type (tall fescue and wheat) for each year. However, some fecal samples were not sufficient for processing; this is reflected on the basis of individual bacterial strains presented in the tables. Except for the calves and the tall fescue group, concentration of TET^r^ *Enterococcus* spp. closely follows that of the total enterococci (generic) population. Similarly, TET^r^ *Enterococcus* spp. prevalence was also high (≥96%) closely following that of the generic population. On the other hand, ERY^r^ *Enterococcus* spp. was quantifiable only from a single animal although the prevalence was over 40% (**Table 1**).

**Table 1:** Concentrations and prevalence of generic- and antibiotic resistant- enterococci by age and treatment group in cow-calf production system.

	Concentration (log_10_/g)						
Outcome	Overall	Age group			Treatment group		
		Calves	Cows	*P*-value	Wheat	Fescue	*P*-value
Generic *Enterococcus* spp.	4.8	4.8	4.8	0.868	4.9	4.7	0.191
TET^r^ *Enterococcus* spp.	4.5	3.2	4.8	<0.001	4.7	4.1	0.032
	**Prevalence (%)**						
	Overall (n=202)	Calves (n=96)	Cows (n=106)	*P*-value	Wheat (n=101)	Fescue (n=101)	*P*-value
Generic enterococci	98.5	97.9	99.1	0.514	97.0	100	0.246^*^
TET^r^ enterococci	96.5	96.9	96.2	0.802	96.0	97.0	0.701
ERY^r^ enterococci	43.1	44.8	41.5	0.638	44.6	41.6	0.670

TET^r^, tetracycline resistant; ERY^r^, erythromycin resistant; *=Fisher’s exact P-value; prevalence was calculated from the total number (n) of the fecal samples presented in parenthesis.

### Effects of age and pasture type on the concentrations and prevalence of generic-, ERY^r^- and TET^r^-*Enterococcus* species

**Figure 1:**
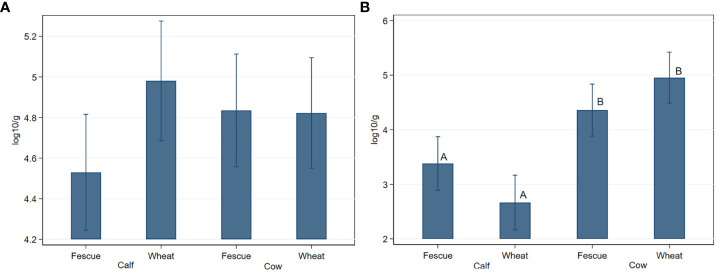
Fecal concentrations of generic- **(A)** and tetracycline resistant- **(B)** *Enterococcus* species in cow-calf production system. When shown, different letters on the bar graphs indicate statistically significant differences at *P* < 0.05. Bar graphs are presented as mean concentrations and their 95% confidence intervals.

**Figure 2:**
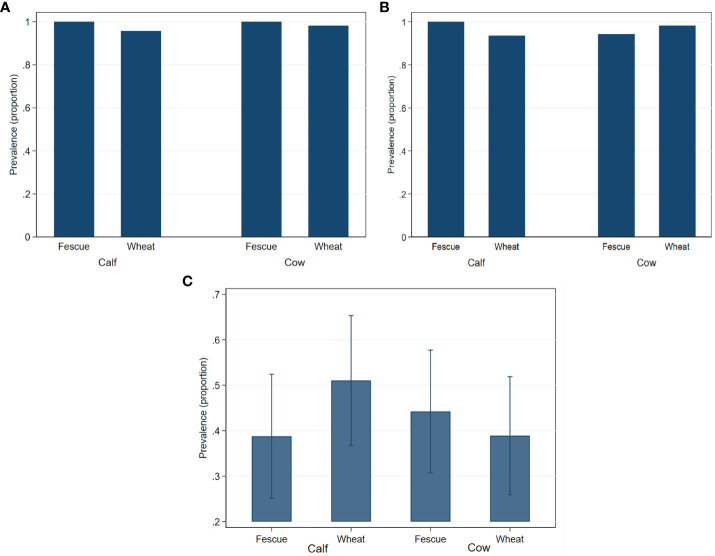
Fecal prevalence of generic- **(A)**, tetracycline resistant- **(B)** and erythromycin resistant- **(C)** *Enterococcus* species in cow-calf production system. Bar graphs are presented as mean prevalence values and their 95% confidence intervals.

Generic enterococci concentration did not significantly (*P*>0.05) differ by age or by pasture type (**Figure 1A**). Concentration of TET^r^-*Enterococcus* spp. were significantly (*P=*0.005) lower in the calves than in the cows, with a greater age effect observed in the wheat group than in the tall fescue due to a significant (*P*=0.008) age by pasture type interaction (**Figure 1B**). ERY^r^ *Enterococcus* spp. was quantifiable at 2.7 logs from a single cow in the fescue group in 2018. The prevalence of generic-, TET^r^-, and ERY^r^ *- Enterococcus* spp. did not significantly (*P*>0.05) differ by age or by pasture type (**Figure 2**).

### Prevalence of *Enterococcus* species detected

Up to two isolates from each positive sample were PCR tested for species identification. Among generic enterococci isolates, seven *Enterococcus* spp. were identified dominated by *E. durans* (38%), followed by *E. mundtii* (22.5%), and *E. faecium* (21.5%), which together accounted for over 80% of the generic enterococci isolate population. Within age and treatment groups, however, there was variation in terms of predominant spp. Six isolates could not be identified by the method used (**Table 2**). The proportion of *E. faecium* isolates obtained from cows that grazed on the wheat pasture was significantly (*P*=0.005) lower than in cows that grazed on tall fescue, with no significant difference in the calves. Proportions of *E. mundtii* isolates obtained from both cows and calves were significantly (*P*<0.001) lower in the wheat pasture than the tall fescue. The proportion of *E. durans* isolates obtained from both cows and calves grazed on wheat was about twice as much than that obtained from tall fescue (*P*<0.001). The *E. casseliflavus* isolates were all obtained from the calves (Fisher’s exact test *P*=0.009).

**Table 2:** Prevalence of *Enterococcus* species among enterococci isolates identified from cow-calf populations, by media type.

Generic enterococci (n=302 isolates)					
Species	Total (n=302)	Calves		Cows	
		Fescue (n=65)	Wheat (n=55)	Fescue (n=93)	Wheat (n=89)
*E. faecalis*	10.9	5.8	11.3	8.8	16.6
*E. faecium*	21.5	13.8 ^A^	23.6 ^AB^	32.3 ^B^	14.6 ^A^
*E. mundtii*	22.5	39.9^B^	11.0 ^A^	31.2 ^B^	7.8 ^A^
*E. casseliflavus*	1.7	3.1	5.5	0	0
*E. durans*	37.8	26.0 ^A^	49.2 ^B^	27.0 ^A^	50.5 ^B^
*E. gallinarum*	0.3	0	0	0	1.1
*E. hirae*	3.3	3.1	1.8	2.2	5.6
Not identified	2.0	4.6	1.8	1.1	1.1
**Tetracycline resistant enterococci (n=352 isolates)**					
Species	Total (n=352)	Calves		Cows	
		Fescue (n=88)	Wheat (n=81)	Fescue (n=87)	Wheat (n=96)
*E. faecalis*	2.0	1.1	4.9	2.3	0
*E. faecium*	53.7	51.1	63.0	51.7	50.0
*E. casseliflavus*	1.7	2.3	0	1.1	3.1
*E. durans*	16.8	19.3^AB^	9.9^A^	9.2^A^	27.1^B^
*E. hirae*	25.0	23.9	21.0	35.6	19.8
Not identified	0.9	2.3	1.2	0	0
**Erythromycin resistant enterococci (n= 111 isolates)**					
Species	Total (n=111)	Calves		Cows	
		Fescue (n=16)	Wheat (n=30)	Fescue (n=33)	Wheat (n=32)
*E. faecalis*	22.5	62.5^B^	3.3^A^	21.2^A^	21.9^A^
*E. faecium*	21.6	12.5^AB^	26.7^AB^	36.4^B^	6.3^A^
*E. casseliflavus*	19.8	0	6.7^A^	33.3^B^	28.1^AB^
*E. durans*	8.1	12.5	13.3	3.0	6.3
E. hirae^*^	5.4	0	13.3	0	6.3
E. avium^*^	7.2	0	10.0	0	15.6
*E. asini*	2.7	0	6.7	0	3.1
Not identified	12.6	12.5	20.0	6.1	12.5

Different superscripted letters indicate significant differences, where shown. *Fisher’s exact test.

TET^r^ enterococci isolates were identified into five spp. with *E. faecium* representing over half (53%) of the population, followed by *E. hirae* (25%), and *E. durans* (17%); the three spp. together accounted for over 95% of the TET^r^ enterococci population. Three TET^r^ enterococci isolates could not be identified into spp. by the method used (**Table 2**). Pasture type had age dependent effect (i.e., interaction) on the prevalence of *E. durans*: the prevalence was significantly (*P*=0.001) greater in the wheat group than the tall fescue group in the cows. Prevalence of the other spp. was not affected by age or treatment group (**Table 2**).

ERY^r^ enterococci were identified into seven spp.; 14 isolates identified by the method used. Top three spp. were *E. faecalis* (22.5%), *E. faecium* (21.6%) and *E. casseliflavus* (19.8%) accounting for approximately 64% of the population (**Table 2**). Pasture type had a significant (*P*= 0.002) age dependent effect on the prevalence of *E. faecalis*; prevalence of *E. faecalis* was greater in the calves that grazed on tall fescue than the remaining groups. Similarly, cows grazed on tall fescue had a significantly (*P*= 0.008) greater prevalence of *E. faecium* than cows grazed on wheat. Prevalence of *E. casseliflavus* isolates was significantly (*P*= 0.027) greater in the tall fescue cows than wheat calves; none was detected from tall fescue calves showing significant age effect (*P*= 0.003) and no significant pasture effect (*P*= 0.939). *E. hirae*, *E. avium* and *E. asini* were detected only in the wheat group (**Table 2**).

### Effect of age and pasture type on the distribution of resistance genes

Seventy percent of TET^r^ enterococci isolates were positive for *tet*(M); *tet*(L) and *tet*(O) represent the remaining 30% (**Table 3**). One TET^r^ *Enterococcus* isolate was negative for all the 11 *tet* genes tested. The proportion of isolates carrying *tet*(M) was significantly (*P*=0.001) greater among the calf isolates, regardless of pasture type, than the isolates obtained from cows that grazed on tall fescue. However, wheat grazing diluted the age effect by increasing the proportion of the isolates carrying *tet*(M) among the cows grazed on wheat, although the difference between the cows grazed on the two pasture types was not statistically different (**Table 3**). Calf TET^r^ enterococci isolates were almost three times (odds ratio =2.8; 95% confidence interval: 1.5-5.3) more likely to carry *tet*(M) compared to the cow isolates adjusted for pasture type and interaction. The prevalence of *tet*(L) was significantly (*P*<0.001) greater among the cows in the fescue group than the wheat groups; on the other hand, that of *tet*(O) was significantly (*P*=0.034) higher among the cows on the wheat group than calves in the tall fescue group (**Table 3**).

**Table 3:** Effects of age and pasture type on the prevalence (%) of tetracycline- and macrolide- resistance genes among tetracycline- and erythromycin- resistant enterococci identified from the feces of cow-calf production system.

Tetracycline resistant enterococci (n=361 isolates)					
Gene	Total (n=361)	Calves		Cows	
		Fescue (n=93)	Wheat (n=81)	Fescue (n=93)	Wheat (n=94)
*tet*(M)	69.8	77.4^B^	75.3^B^	54.8^A^	72.3^AB^
*tet*(L)	19.1	18.3^AB^	12.3^A^	34.4^B^	10.6^A^
*tet*(O)	10.3	3.2^A^	12.3^AB^	10.8^AB^	14.9^B^
*tet*(S)	0.6	0	0	0	2.1
**Erythromycin resistant enterococci (n=113 isolates)**					
Gene	Total (n=113)	Calves		Cows	
		Fescue (n=11)	Wheat (n=34)	Fescue (n=34)	Wheat (n=34)
*erm*(B)	84.1	72.7	91.2	76.5	88.2
*msr*(C)	11.5	18.2	5.9	23.5	2.9
*mef*(A)	1.2	0	0	0	5.9
*erm*(Q)	0.9	9.1	0	0	0

Different superscripted letters indicate significant differences, where shown.

Overwhelming majority (84%) of ERY^r^ isolates carried *erm*(B). The prevalence of *erm*(B) was significantly (*P*=0.047) higher among the wheat isolates than the fescue group. On the other hand, the prevalence of *msr*(C) was significantly (*P*=0.006) higher in the fescue isolates than in the wheat isolates. While *mef*(A) gene was detected only in cows that grazed wheat, *erm*(Q) was detected only in the calves that grazed on tall fescue (**Table 3**). Two ERY^r^ isolates were not positive for the genes targeted.

### Distribution of resistance genes by *Enterococcus* species

Among TET^r^ enterococci isolates, over 50% (range: 50-95%) of the isolates in all species but *E. hirae* carried *tet*(M). However, 85% of *E. hirae* isolates carried either *tet*(L) or *tet*(O). The two *tet*(S) positive isolates were *E. casseliflavus* (**Table 4**). While all minor *Enterococcus* spp. harbored *erm*(B), 10% of *E. faecalis*, and 39% of *E. faecium* isolates were positive for *msr*(C) (**Table 4**).

**Table 4:** Prevalence (%) of tetracycline- and macrolide- resistance genes, respectively among phenotypically tetracycline- and erythromycin- resistant *Enterococcus* species obtained from the feces of beef cow-calf production.

	% Of tetracycline resistance genes					% Of erythromycin resistance genes			
Species	No. of isolates	*tet*(M)	*tet*(L)	*tet*(O)	*tet*(S)	Species	No. of isolates	*erm*(B)	*msr*(C)
*E. faecalis*	7	71.4	28.6	0	0	*E. faecalis*	20	90	10
*E. faecium*	179	95.0	4.5	0.6	0	*E. faecium*	23	60.9	39.1
*E. casseliflavus*	6	50.0	16.7	0	33.3	*E. casseliflavus*	22	100	0
*E. durans*	59	86.4	5.1	8.5	0	*E. durans*	9	100	0
*E. hirae*	86	15.1	54.7	30.2	0	*E. hirae*	6	83.3	0
						*E. asini*	3	100	0
						*E. avium*	8	100	0

A single erm(Q) positive isolate and the two mef(A) positive isolates were not identified to species.

One *E. hirae* isolate was negative for all resistance genes tested.

## Discussion

In the adult beef cows, the concentration and prevalence of TET^r^ enterococci were closely similar to that of the generic enterococci population suggesting the widespread occurrence and abundance of TET^r^ enterococci population in beef cows. On the other hand, the prevalence of ERY^r^ enterococci was 42%. Erythromycin resistant enterococci was enumerable only from a single animal suggesting that ERY^r^ enterococci occur at a low concentration in the absence of or low antibiotic selection pressure under extensive animal production such as cow-calf operation. This phenomenon of low abundance (enumerable from only five animals) but high prevalence (69%) was previously reported from a beef cow population. (Agga et al., 2016) Unlike dairy cattle production, in cow-calf operation calves comingle with the cows until weaning, adult cows can potentially transmit ARB to the calves, as shown in the present study by similar prevalence of both TET^r^- and ERY^r^- enterococci in the calves and cows (**Table 1**).

Once in the feedlot, the level of resistant bacteria would increase (Call et al., 2008), with subsequent carcass contamination; ERY^r^- enterococci were detected from 88% of colon fecal samples of beef cattle at slaughter. (Vikram et al., 2017) When culled cows are sold and processed, ground beef contamination can occur. ERY^r^- enterococci were detected from 38% and 48% of organic and conventional retail ground beef claims, respectively. (Schmidt et al., 2021) A large retrospective longitudinal analysis of ground beef obtained from retail markets in the USA showed 92.7% enterococcal contamination (Tyson et al., 2018).

Although the prevalence of TET^r^- and ERY^r^- enterococci did not significantly differ, concentration of TET^r^ enterococci was significantly higher in the cows than in the calves. Several studies indicated that the level of ARB decreases with age of the animal. (Call et al., 2008; Gaire et al., 2021) Evaluating the age effect requires a longitudinal study of following a cohort of calves over time. Since our study was cross sectional, the study could not evaluate age effect within the cohorts of the calves. Rather, this study compared the level of ARB between the calf and cow populations that were kept together. Monitoring the status of ARB in beef calves and the breeding cows with periodic fecal sampling and testing would help answer the age effect and define the baseline level in calves prior to entering the feedlot. Furthermore, previous studies reporting age effect were conducted in *E. coli*. Therefore, our study can be used as a baseline for Gram-positive bacteria such as enterococci population in beef cattle production.

Wheat grazing at weaning tends to reduce the concentration of TET^r^ enterococci in the calves compared to cows grazing on wheat. The mechanisms and benefits to calves grazing on wheat need to be explored for the mitigation of AMR, especially because calves go to feedlot for beef production, with food safety implication. However, we previously reported from the same cow-calf population that wheat grazing had no effect on the prevalence and concentration of TET^r^ *E. coli* (Agga et al., 2022b) suggesting the differential effect of wheat grazing on Gram-positive and Gram-negative bacteria.

The top three *Enterococcus* species identified among the generic- (*E. drans, E. mundtii followed by E. faecium*), ERY^r^- (*E. faecalis, E. faecium and E. casseliflavus*) and TET^r^- (*E. faecium, E. hirae and E. durans*) isolates (**Table 2**) represent the most frequently identified species from the GIT of humans and animals (Aarestrup et al., 2002; Dec et al., 2019). These top six species (*E. faecium* was shared among the three media types; *E. durans* was shared between generic and TET^r^ isolates) are among the most frequently identified retail meat isolates (Tyson et al., 2018), signifying their foodborne importance (Murray et al., 2022). Among the ground beef isolates, ERY- (2.4% in *E. faecalis*, and 7.5% in *E. faecium* isolates) and TET- (23% in *E. faecalis*, and 25% in *E. faecium* isolates) resistance was observed relatively less frequently compared to other retail meat types (Tyson et al., 2018). The top species isolated from the cow-calf population are also among the species isolated from cattle feces and feedlot environments, and cow-calf operations. (USDA, 2012; Zaheer et al., 2020) Furthermore, TET and macrolide resistance were the most prevalent phenotypes in *E. hirae*, *E. faecalis*, and *E. faecium* isolates obtained from feedlot cattle. (Zaheer et al., 2020) The present study notes that TET^r^- isolates were less diverse than generic and ERY^r^ isolates, with fewer number of species identified and dominated by three species which accounted for 95% of the total TET^r^ isolates (**Table 2**).

Although age modified the effect of wheat grazing on the species distribution, *E. casseliflavus* was the only species that was detected at a significantly higher prevalence in the calves than the cows. The reason behind this species differential is unknown, but *E. casseliflavus* is among the most commonly isolated species, together with *E. faecalis* and *E. faecium* (Aarestrup et al., 2002), from insects which aid in the spread of enterococcal species (Macovei and Zurek, 2006). Another significant finding of the current study is the potential of wheat grazing to modify the gut microbiota. Wheat grazing significantly reduced the prevalence of generic- and ERY^r^ *- E. faecium* (in the cows), generic *E. mundtii* (in both cows and calves), ERY^r^ *E. faecalis* (calves). On the other hand, wheat grazing significantly increased the prevalence of both generic- (doubled in the wheat group) and TET^r^- *E. durans*; ERY^r^- *E. hirae*, -*E. avium*, and -*E. asini* were detected only from the wheat group. The findings suggest that wheat grazing modifies the microbiota of the gut, either by increasing or decreasing the proportion of specific bacterial species. The mechanisms for this effect and potential adaptation of wheat grazing to mitigate the major enterococci species, *E. faecium* and *E. faecalis*, in beef calf production need to be further investigated.

Tetracycline- and macrolide- resistance of the enterococci isolates obtained from the cow-calf population were conferred exclusively by *tet*(M), *tet*(L) and *tet*(O), and *erm*(B) and *msr*(C), respectively as reported in the literature. (Frye and Jackson, 2013; Agga et al., 2022c) However, the distribution of the three *tet* genes is more diverse in the cows than in the calves, where *tet*(M) predominates. The age dependence for *tet*(M) in conferring TET resistance in enterococci indicates the unique role that *tet*(M) plays to counter antibiotic selection pressure in the calves which require antibiotic treatments than the breeding beef cows due to bacterial infections before weaning age. Beef cows on the other hand are less susceptible to infections due to less physiologic demand as well as less infection density, as opposed to dairy cattle, thus do not require much antibiotic treatment, and mass therapy is not common (Call et al., 2008; USDA, 2012). Much like the species distribution, wheat grazing affected the distribution of the *tet* and MLS_B_ genes by either increasing [*tet*(O), *erm*(B)] or decreasing [*tet*(L), *msr*(C)] their prevalence. Wheat grazing had a similar effect on resistance gene distribution among TET^r^ and third generation cephalosporin resistant *E. coli* isolates characterized from the same cow-calf populations (Agga et al., 2022b). These findings suggest that wheat grazing significantly affects the resistance gene distribution by favoring the expansion of bacterial population carrying certain genes, while suppressing bacterial population carrying other genes despite conferring the same phenotypic resistance.

## Conclusions

The study detected erythromycin, a macrolide antibiotic categorized as high priority critically important for medical use, resistant *Enterococcus* species of significant public health importance in two-fifths of the fecal samples obtained from cow-calf production system. Tetracycline resistant *Enterococcus* species were abundant and widespread in the cow-calf populations. The study reported that wheat grazing, either alone or through its interaction with the age of the animal, affected the abundance of TET^r^ enterococci, enterococcal species and resistance gene distributions. Calves had a significantly lower abundance of tetracycline resistant enterococci, and *tet*(M) plays a major role in conferring tetracycline resistance among the calf isolates, with more diverse tetracycline resistance genes being detected in the cows. The study suggests that AMR can persist in food animal production systems with less antibiotic selective pressure such as cow-calf production. Further studies are needed to harness the dual benefit of wheat grazing to improve beef calf growth performance, and as a potential mitigation strategy to modify pathogenic and antimicrobial resistant bacteria pathogenic to humans such as enterococci prior to feedlot operation.

## Data availability statement

The original contributions presented in the study are included in the article/**Supplementary Material**. Further inquiries can be directed to the corresponding author.

## Ethics statement

The animal study was reviewed and approved by Western Kentucky University’s Institutional Animal Care and Use Committee (IACUC# 17-09).

## Author contributions

GA conceptualized the study, acquired funding, led the investigation, analyzed the data and wrote the manuscript. HG was involved in conceptualization, funding acquisition and supervising animal management. AN was involved in conceptualization, funding acquisition and field study. All authors contributed to the article and approved the submitted version.

## Funding

This research was funded by the U.S. Department of Agriculture, Agricultural Research Service (Project No. 5040-12630-006-00D) and through a USDA-WKU cooperative agreement (Project Number: 5040-12630-006-30-S).

## Acknowledgments

We thank Rohan Parekh for technical support. Mention of trade names or commercial products in this publication is solely for the purpose of providing specific information and does not imply recommendation or endorsement by the U.S. Department of Agriculture. USDA is an equal opportunity provider and employer.

## Conflict of interest

The authors declare that the research was conducted in the absence of any commercial or financial relationships that could be construed as a potential conflict of interest.

## Publisher’s note

All claims expressed in this article are solely those of the authors and do not necessarily represent those of their affiliated organizations, or those of the publisher, the editors and the reviewers. Any product that may be evaluated in this article, or claim that may be made by its manufacturer, is not guaranteed or endorsed by the publisher.

## Supplementary material

Supplementary Figure 1 Representative gel images from capillary electrophoresis for enterococci genus confirmation and species identification from cow-calf production system. The molecular sizes are approximated base pairs (bp). Genus= genus specific gene marker; FL= *E. faecalis*; FM= *E. faecium*; DU= *E. durans*; CA= *E. casseliflavus;* GA= *E. gallinarum;* MU= *E. mundtii;* AV= *E. avium;* HI= *E. hirae;* AS= *E. asini.*

Supplementary Figure 2 Representative gel images from capillary electrophoresis for tetracycline resistance genes from enterococci isolated from cow-calf production system. The molecular sizes are approximated base pairs (bp).

Supplementary Figure 3 Representative gel images from capillary electrophoresis for macrolide resistance genes from enterococci isolated from cow-calf production system. The molecular sizes are approximated base pairs (bp).

Supplementary Table 1 Primers used for PCR speciation of enterococci isolates obtained from the feces of cows and pre-weaned calves in cow-calf herds (Jackson et al., 2004).

Supplementary Table 2 Primers used for PCR detection of tetracycline resistance *(tet)* genes from phenotypically tetracycline resistant *Enterococcus* species isolated from the feces of cows and pre-weaned calves in cow-calf herds.

Supplementary Table 3 Primers used for PCR detection of macrolide resistance genes from phenotypically erythromycin resistant *Enterococcus* species isolated from the feces of cows and pre-weaned calves in cow-calf herds.
