# Supplementary figures and images for: Effects of age and pasture type on the concentration and prevalence of tetracycline and macrolide resistant Enterococcus species in beef cow-calf production system
Source: Front Antibiot. 2022 Nov 3;1:1052316. doi: 10.3389/frabi.2022.1052316 (PMC11733798; doi:10.3389/frabi.2022.1052316)

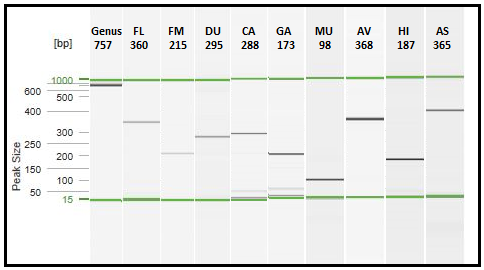

Supplement: Supplementary Figure 1 — Representative gel images from capillary electrophoresis for enterococci genus confirmation and species identification from cow-calf production system. The molecular sizes are approximated base pairs (bp). Genus= genus specific gene marker; FL= E. faecalis; FM= E. faecium; DU= E. durans; CA= E. casseliflavus; GA= E. gallinarum; MU= E. mundtii; AV= E. avium; HI= E. hirae; AS= E. asini. [file Image_1.tif]

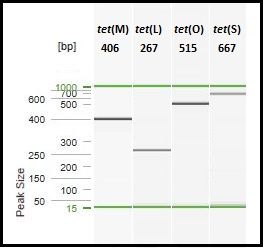

Supplement: Supplementary Figure 2 — Representative gel images from capillary electrophoresis for tetracycline resistance genes from enterococci isolated from cow-calf production system. The molecular sizes are approximated base pairs (bp). [file Image_2.tif]

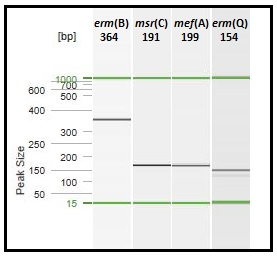

Supplement: Supplementary Figure 3 — Representative gel images from capillary electrophoresis for macrolide resistance genes from enterococci isolated from cow-calf production system. The molecular sizes are approximated base pairs (bp). [file Image_3.tif]
