# Supplementary material for: Effects of age and pasture type on the concentration and prevalence of tetracycline and macrolide resistant Enterococcus species in beef cow-calf production system
Source: Front Antibiot. 2022 Nov 3;1:1052316. doi: 10.3389/frabi.2022.1052316 (PMC11733798; doi:10.3389/frabi.2022.1052316)
Supplement: Supplementary Table 1 — Primers used for PCR speciation of enterococci isolates obtained from the feces of cows and pre-weaned calves in cow-calf herds (Jackson et al., 2004). [file Table_1.pdf]

Supplementary Table 1: Primers used for PCR speciation of enterococci isolates obtained from the feces of cows and pre-weaned calves in cow-calf herds (Jackson et al., 2004).

Species	Primer	Primer sequence (5'-3')	Tm (°C)	Product size (bp)	Multiplex group
Genus specific	El	TCA ACC GGG GAG GGT	55 or 60	757	(Deasy et al., 2000)
	E2	ATT ACT AGC GAT TCC GG			
faecalis	FL1	ACTTATGTGACTAACTTAACC	55	360	1
	FL2	TAATGGTGAATCTTGGTTTGG			
faecium	FM1	GAAAAAACAATAGAAGAATTAT		215	
	FM2	TGCTTTTTTGAATTCTTCTTTA			
durans	DU1	CCTACTGATATTAAGACAGCG		295	
	DU2	TAATCCTAAGATAGGTGTTTG			
malodoratus	MA1	GTAACGAACTTGAATGAAGTG		134	
	MA2	TTGATCGCACCTGTTGGTTTT			
casseliflavus	CA1	TCCTGAATTAGGTGAAAAAAC	55	288	2
	CA2	GCTAGTTTACCGTCTTTAACG			
gallinarum	GA1	TTACTTGCTGATTTTGATTCG		173	
	GA2	TGAATTCTTCTTTGAAATCAG			
solitarius	SO1	AAACACCATAACACTTATGTGACG		371	
	SO2	AATGGAGAATCTTGGTTTGGCGTC			
dispar	DI1	GAACTAGCAGAAAAAAGTGTG	60	284	3
	DI2	GATAATTTACCGTTATTTACC			
pseudoavium	PV1	TCTGTTGAGGATTTAGTTGCA		173	
	PV2	CCGAAAGCTTCGTCAATGGCG			
saccharolyticus	SA1	AAACACCATAACACTTATGTG		371	
	SA2	GTAGAAGTCACTTCTAATAAC			
flavescens	FV1	GAATTAGGTGAAAAAAAAGTT	60	284	4
	FV2	GCTAGTTTACCGTCTTTAACG			
mundtii	MU1	CAGACATGGATGCTATTCCATCT		98	
	MU2	GCCATGATTTTCCAGAAGAAT			
sulfureus	SU1	TCAGTGGAAGACTTAATCGCA		173	
	SU2	CCAAATGTATCTTCGATCGCT			
avium	AV1	GCTGCGATTGAAAAATATCCG	55	368	5
	AV2	AAGCCAATGATCGGTGTTTTT			
columbae	CO1	GAATTTGGTACCAAGACAGTT		284	
	CO2	GCTAATTTACCGTTATCGACT			
seriolicida	SE1	ACACAATGTTCTGGGAATGGC		100	
	SE2	AAGTCGTCAAATGAACCAAAA			
cecorum	CE1	AAACATCATAAAACCTATTTA	55	371	6

	CE2	AATGGTGAATCTTGGTTCGCA			
raffinosus	RF1	GTCACGAACTTGAATGAAGTT		287	
	RF2	AATGGGCTATCTTGATTCGCG			
hirae	HI1	CTTTCTGATATGGATGCTGTC		187	
	HI2	TAAATTCTTCCTTAAATGTTG			
asini	AS1	GCATCATGACAAGCATCACGC	60	365	7
	AS2	GGCTTTTTGCCTTCAGATAAA			
gilvus	GI1	CTGGCTGGGCTTGGCTAGTGA		98	
	GI2	ATAATCGGTGTTTTACCGTCT			
pallens	PA1	TGGCACCAAATGCTGGCGGAA		160	
	PA2	TGGTGTAGAAGTAATTTCAAG			
porcinus/villorum	PO1	TGGTTTCTGATATGGATGCGA		280	
	PO2	GTAATCGCTAATTTCTCTCCA			

Supplementary Table 2: Primers used for PCR detection of tetracycline resistance (*tet*) genes from phenotypically tetracycline resistant *Enterococcus* spp. isolated from the feces of cows and pre-weaned calves in cow-calf herds.

Gene	Primer	Primer sequence (5'-3')	Tm (°C)	Product size (bp)	Reference
tet(M)	F	GTG GAC AAA GGT ACA ACG AG	55.0	406	(Ng et al., 2001)
	R	CGG TAA AGT TCG TCA CAC AC			
tet(L)	F	TCG TTA GCG TGC TGT CAT TC	55.0	267	(Ng et al., 2001)
	R	GTA TCC CAC CAA TGT AGC CG			
tet(O)	F	AAC TTA GGC ATT CTG GCT CAC	55.0	515	(Ng et al., 2001)
	R	TCC CAC TGT TCC ATA TCG TCA			
tet(P)	F	CTT GGA TTG CGG AAG AAG AG	55.0	676	(Ng et al., 2001)
	R	ATA TGC CCA TTT AAC CAC GC			
tet(S)	F	CAT AGA CAA GCC GTT GAC C	55.0	667	(Ng et al., 2001)
	R	ATG TTT TTG GAA CGC CAG AG			
tet(D)	F	AAA CCA TTA CGG CAT TCT GC	55.0	787	(Ng et al., 2001)
	R	GAC CGG ATA CAC CAT CCA TC			
tet(G)	F	GCT CGG TGG TAT CTC TGC TC	55.0	844	(Ng et al., 2001)
	R	AGC AAC AGA ATC GGG AAC AC			
tet(Q)	F	AGA ATC TGC TGT TTG CCA GTG	63.0	169	(Aminov et al., 2001)
	R	CGG AGT GTC AAT GAT ATT GCA			
tet(W)	F	GAG AGC CTG CTA TAT GCC AGC	64.0	168	(Aminov et al., 2001)
	R	GGG CGT ATC CAC AAT GTT AAC			
tet(X)	F	CAA TAA TTG GTG GTG GAC CC	55.0	468	(Ng et al., 2001)
	R	TTC TTA CCT TGG ACA TCC CG			
tet(K)	F	TCG ATA GGA ACA GCA GTA	55.0	169	(Ng et al., 2001)
	R	CAG CAG ATC CTA CTC CTT			

Supplementary Table 3: Primers used for PCR detection of macrolide resistance genes from phenotypically erythromycin resistant *Enterococcus* spp. isolated from the feces of cows and pre-weaned calves in cow-calf herds.

Gene	Primer	Primer Sequence (5'-3')	Tm (°C)	Product size (bp)	Reference
erm(A)	F	AGTCAGGCTAAATATAGCTATC	63.0	157	(Koike et al., 2010)
	R	CAAGAACAATCAATACAGAGTCTAC			
erm(B)	F	GAT ACC GTT TAC GAA ATT GG	58.0	364	(Chen et al., 2007)
	R	GAA TCG AGA CTT GAG TGT GC			
erm(C)	F	AATCGTGGAATACGGGTTTGC	62	293	(Koike et al., 2010)
	R	CGTCAATTCCTGCATGTTTTAAGG			
msr(C)	F	TCGTTTTGTCATGAGACAAACAG	58.0	191	(Beukers et al., 2015)
	R	AAATTAGTCGGTTCATCTAACAG			
mef(A)	F	GGAGCTACCTGTCTGGATGG	58.0	199	(Szczepanowski et al., 2009)
	R	CAACCGCCGGACTAACAATA			
mef(E)	F	CCTGCAAATGGCGATTATTT	58.0	199	(Szczepanowski et al., 2009)
	R	AATAGCAAGCACTGCACCAG			
erm(Q)	F	CACCAACTGATATGTGGCTAG	68-60	154	(Koike et al., 2010)
	R	CTAGGCATGGGATGGAAGTC			
msr(D)	F	TTGGACGAAGTAACTCTG	50.0	370	(Daly et al., 2004)
	R	GCTTGGCTCTTACGTTC			
erm(T)	F	CATATAAATGAAATTTTGAG	51.0	369	(Chen et al., 2007)
	R	ACGATTTGTATTTAGCAACC			
cfr	F	TGAAGTATAAAGCAGGTTGGGAGTCA	55	~750	(Brenciani et al., 2016)
	R	ACCATATAATTGACCACAAGCAGC			
cfr(B)	F	CAGGAGACGAAAAAATAGAAAC	55	398	(Brenciani et al., 2019)
	R	AAAAGGAGAATGTAATGAAAATGT			
optr(A)	F	TACTTGATGAACCTACTAACCA	55	422	(Brenciani et al., 2016)

	R	CCTTGAACTACTGATTCTCGG			
poxt(A)	F	GAACGCTTGGAGTATTTCGACTTC	55	778	(Brenciani et al., 2019)
	R	CTGGACTGAGAATACCCATC			
eat(A)	F	GCAATCGTGAATCGGATGG	55	1068	(Isnard et al., 2013)
	R	GGAATCGTACAGCGAACGC			

References

Aminov, R.I., Garrigues-Jeanjean, N., and Mackie, R.I. (2001). Molecular ecology of tetracycline resistance: development and validation of primers for detection of tetracycline resistance genes encoding ribosomal protection proteins. *Appl Environ Microbiol* 67(1), 22-32. doi: 10.1128/AEM.67.1.22-32.2001.

Beukers, A.G., Zaheer, R., Cook, S.R., Stanford, K., Chaves, A.V., Ward, M.P., et al. (2015). Effect of in-feed administration and withdrawal of tylosin phosphate on antibiotic resistance in enterococci isolated from feedlot steers. *Front Microbiol* 6, 483. doi: 10.3389/fmicb.2015.00483.

Brenciani, A., Fioriti, S., Morroni, G., Cucco, L., Morelli, A., Pezzotti, G., et al. (2019). Detection in Italy of a porcine *Enterococcus faecium* isolate carrying the novel phenicol-oxazolidinone-tetracycline resistance gene poxtA. *Journal of Antimicrobial Chemotherapy*.

Brenciani, A., Morroni, G., Pollini, S., Tiberi, E., Mingoia, M., Varaldo, P.E., et al. (2016). Characterization of novel conjugative multiresistance plasmids carrying cfr from linezolid-resistant *Staphylococcus epidermidis* clinical isolates from Italy. *Journal of Antimicrobial Chemotherapy* 71(2), 307-313.

Chen, J., Yu, Z., Michel, F.C., Jr., Wittum, T., and Morrison, M. (2007). Development and application of real-time PCR assays for quantification of *erm* genes conferring resistance to macrolides-lincosamides-streptogramin B in livestock manure and manure management systems. *Appl Environ Microbiol* 73(14), 4407-4416. doi: 10.1128/AEM.02799-06.

Daly, M.M., Doktor, S., Flamm, R., and Shortridge, D. (2004). Characterization and prevalence of MefA, MefE, and the associated *msr* (D) gene in *Streptococcus pneumoniae* clinical isolates. *Journal of clinical microbiology* 42(8), 3570-3574.

Deasy, B.M., Rea, M.C., Fitzgerald, G.F., Cogan, T.M., and Beresford, T.P. (2000). A rapid PCR based method to distinguish between *Lactococcus* and *Enterococcus*. *Syst Appl Microbiol* 23(4), 510-522. doi: 10.1016/S0723-2020(00)80025-9.

Isnard, C., Malbruny, B., Leclercq, R., and Cattoir, V. (2013). Genetic basis for in vitro and in vivo resistance to lincosamides, streptogramins A, and pleuromutilins (LSAP phenotype) in *Enterococcus faecium*. *Antimicrobial agents and chemotherapy* 57(9), 4463-4469.

Jackson, C.R., Fedorka-Cray, P.J., and Barrett, J.B. (2004). Use of a genus-and species-specific multiplex PCR for identification of enterococci. *Journal of clinical microbiology* 42(8), 3558-3565.

Koike, S., Aminov, R.I., Yannarell, A.C., Gans, H.D., Krapac, I.G., Chee-Sanford, J.C., et al. (2010). Molecular ecology of macrolide-lincosamide-streptogramin B methylases in waste lagoons and subsurface waters associated with swine production. *Microb Ecol* 59(3), 487-498. doi: 10.1007/s00248-009-9610-0.

Ng, L.K., Martin, I., Alfa, M., and Mulvey, M. (2001). Multiplex PCR for the detection of tetracycline resistant genes. *Mol Cell Probes* 15(4), 209-215. doi: 10.1006/mcpr.2001.0363.

Szczepanowski, R., Linke, B., Krahn, I., Gartemann, K.H., Gutzkow, T., Eichler, W., et al. (2009). Detection of 140 clinically relevant antibiotic-resistance genes in the plasmid metagenome of wastewater treatment plant bacteria showing reduced susceptibility to selected antibiotics. *Microbiology* 155(Pt 7), 2306-2319. doi: 10.1099/mic.0.028233-0.
